# Differential expression analysis of genes and long non-coding RNAs associated with KRAS mutation in colorectal cancer cells

**DOI:** 10.1038/s41598-022-11697-5

**Published:** 2022-05-13

**Authors:** Mahsa Saliani, Razieh Jalal, Ali Javadmanesh

**Affiliations:** 1 Department of Chemistry, Faculty of Science, Ferdowsi University of Mashhad, Mashhad, 9177948974 Iran; 2 Novel Diagnostics and Therapeutics Research Group, Institute of Biotechnology, Ferdowsi University of Mashhad, Mashhad, 9177948974 Iran; 3 Department of Animal Science, Faculty of Agriculture, Ferdowsi University of Mashhad, Mashhad, Iran; 4 Stem Cell Biology and Regenerative Medicine Research Group, Research Institute of Biotechnology, Ferdowsi University of Mashhad, Mashhad, 9177948974 Iran

**Keywords:** Biochemistry, Cancer, Cell biology, Computational biology and bioinformatics

## Abstract

KRAS mutation is responsible for 40–50% of colorectal cancers (CRCs). RNA-seq data and bioinformatics methods were used to analyze the transcriptional profiles of KRAS mutant (mtKRAS) in comparison with the wild-type (wtKRAS) cell lines, followed by *in-silico* and quantitative real-time PCR (qPCR) validations. Gene set enrichment analysis showed overrepresentation of KRAS signaling as an oncogenic signature in mtKRAS. Gene ontology and pathway analyses on 600 differentially-expressed genes (DEGs) indicated their major involvement in the cancer-associated signal transduction pathways. Significant hub genes were identified through analyzing PPI network, with the highest node degree for PTPRC. The evaluation of the interaction between co-expressed DEGs and lncRNAs revealed 12 differentially-expressed lncRNAs which potentially regulate the genes majorly enriched in Rap1 and RAS signaling pathways. The results of the qPCR showed the overexpression of PPARG and PTGS2, and downregulation of PTPRC in mtKRAS cells compared to the wtKRAS one, which confirming the outputs of RNA-seq analysis. Further, significant upregualtion of miR-23b was observed in wtKRAS cells. The comparison between the expression level of hub genes and TFs with expression data of CRC tissue samples deposited in TCGA databank confirmed them as distinct biomarkers for the discrimination of normal and tumor patient samples. Survival analysis revealed the significant prognostic value for some of the hub genes, TFs, and lncRNAs. The results of the present study can extend the vision on the molecular mechanisms involved in KRAS-driven CRC pathogenesis.

## Introduction

According to the GLOBOCAN database, colorectal cancer (CRC) is known as one of the most prevalent malignancies with 9.2% of mortality in 2018, which ranks as the second leading cause of the cancer-induced mortality worldwide^1^. It has been estimated that 45% of sporadic CRCs frequently arises from preneoplastic lesions through KRAS proto-oncogene mutation^2^. It encodes a small guanosine triphosphate (GTP)/guanosine diphosphate (GDP) binding protein involved in regulating many cellular responses to several extracellular stimuli by activating over 20 known downstream effectors^3,4^. In addition, the RAS-specific guanine nucleotide exchange factors (GEFs) and guanosine triphosphate hydrolase (GTPase)-activating proteins (GAPs) tightly regulate cycling between an inactive GDP-bound (“off”) and an active GTP-bound (“on”) states of wild-type KRAS (wtKRAS) protein^5,6^. Mutation in KRAS proto-oncogene results in conformational changes, leading to its insensitivity to GAPs and many different effects such as cell growth promotion, cell transformation, angiogenesis, migration, and apoptosis suppression^7^. Therefore, KRAS mutation in CRCs is associated with more reduced survival, increased tumor aggressiveness, and resistance to anti-epidermal growth factor receptor (EGFR) targeted-therapies^8,9^. Following the discovery of an association between the mutant forms of RAS protein with human cancers, numerous studies have been conducted on inhibiting oncogenic RAS signaling for cancer treatment^10,11^. So far, the specific inhibition of oncogenic RAS has not been clinically approved despite extensive efforts on understanding the mechanisms of regulation, intracellular trafficking, and RAS protein signaling activity. Due to the high prevalence and mortality of KRAS mutant CRCs, studying potential molecular biomarkers and mechanisms to improve patient outcomes is becoming increasingly important.

The hundreds of genes, long non-coding RNAs (lncRNAs), and microRNAs (miRs) involved in various signaling pathways, molecular functions, and biological processes have been identified through applying the gene microarray and next-generation RNA sequencing (RNA-seq) which are high-throughput platforms for analyzing gene expression^12,13^. Somatic mutations cause a series of downstream, secondary alterations in the transcriptome, leading to the large-scale disturbances of transcriptional profiles in most cancers^14,15^. For instance, microarray analysis of CRCs with KRAS and EGFR mutations, demonstrated that mutation status leads to the dysregulated transcriptional landscape of the cells^16^. Further, the assessment of differential gene expression signatures revealed an alteration in the signaling pathways such as Wnt, NF-kappa B, and the TGF-beta, and metabolic ones, along with changing the KRAS-related signaling pathways, between CRCs with and without KRAS mutations^17,18^. LncRNAs and miRs, as new and fundamental transcriptional and post-transcriptional regulators with a pivotal role in tumorigenesis and malignant tumor metastasis, have been studied widely^19,20^. Furthermore, the contribution of 282 lncRNAs to CRC heterogeneity has been recognized by monitoring the expression of 4898 lncRNA genes across 566 CRC samples^14^. Some researchers analyzed differential miR expression in colorectal tumors with KRAS and BRAF mutations^15,21^. Activating mutations in KRAS changes the transcriptional profiles of CRC cells significantly. Thus, the further characterization of CRC exploring all specific molecular and cellular aberrations, along with KRAS mutation, seems to be critical for detecting specific therapeutic opportunities^17^.

In the present study, RNA-seq data were downloaded from the Sequence Read Archive database (SRA) to identify the genes and lncRNAs having differential expression between mtKRAS and wtKRAS CRC cell lines. For this purpose, gene ontology (GO), pathway enrichment, and protein–protein interaction (PPI) analyses, as well as evaluating the interaction between co-expressed DEGs and differentially-expressed lncRNAs (DElncRNAs) were conducted. Additionally, the survival analyses of hub genes and transcription factors (TFs) were performed to estimate their prognostic performance as potential biomarkers in CRC patients. The results of qPCR confirmed the outputs of the bioinformatics analysis. In this regard, the expression pattern of the hub DEGs including PPARG, PTGS2, and PTPRC in mtKRAS cells compared to the wtKRAS sample, showed the overexpression of PPARG and PTGS2 and the downregulation of PTPRC both in qPCR and RNA-seq results. The pivotal hub genes (mainly PPARG, PTGS2, PTPRC, CDKN2A, and PRKACB) and lncRNAs (e.g., OGFRL1, DGCR5, and LINC01842) have been previously reported to have critical roles in the tumorigenesis of many cancers harboring KRAS mutation. Finally, the results provide novel insights into the potential biomarkers for KRAS mutant CRC, which may contribute to a better understanding of the molecular and cellular mechanisms mediating the KRAS mutation-induced malignant transformation.

## Results

### Identification of differentially expressed genes and non-coding RNAs

**Figure 1:**
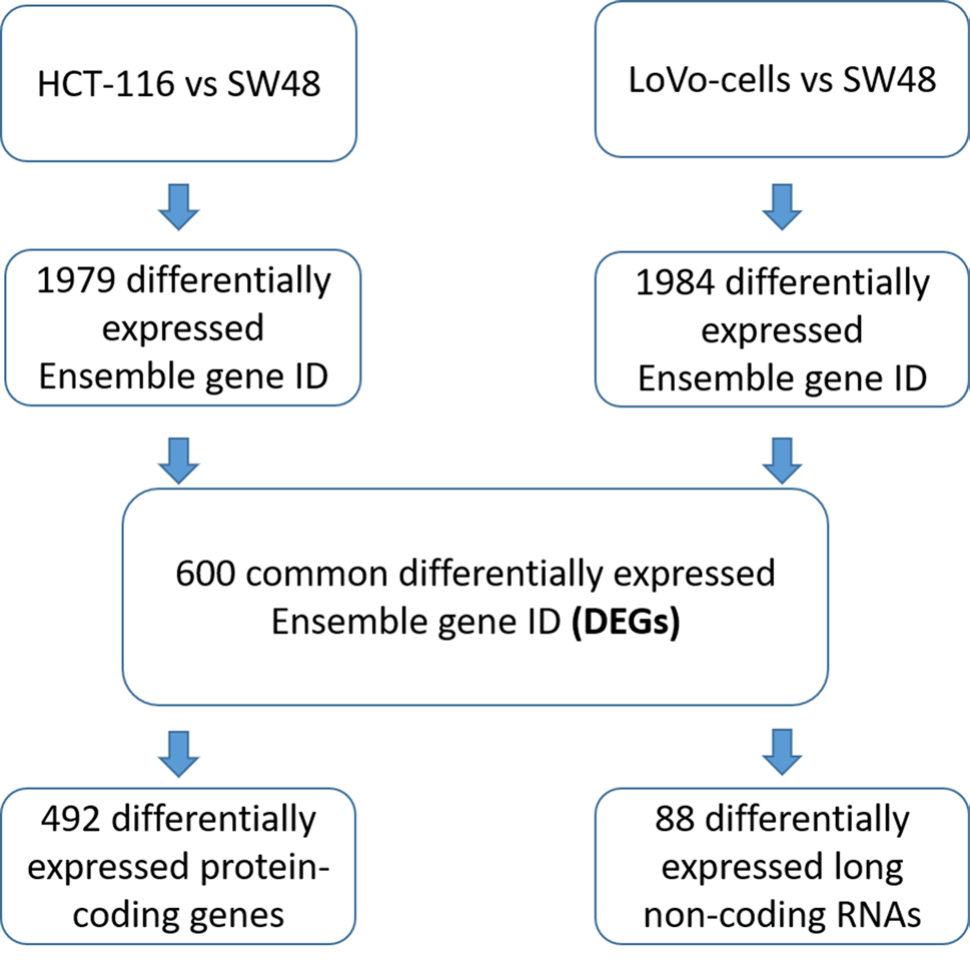
Design of the study. Schematic diagram for a multi-step strategy to identify DEGs and DEncRNAs associated with the KRAS mutation.

**Figure 2:**
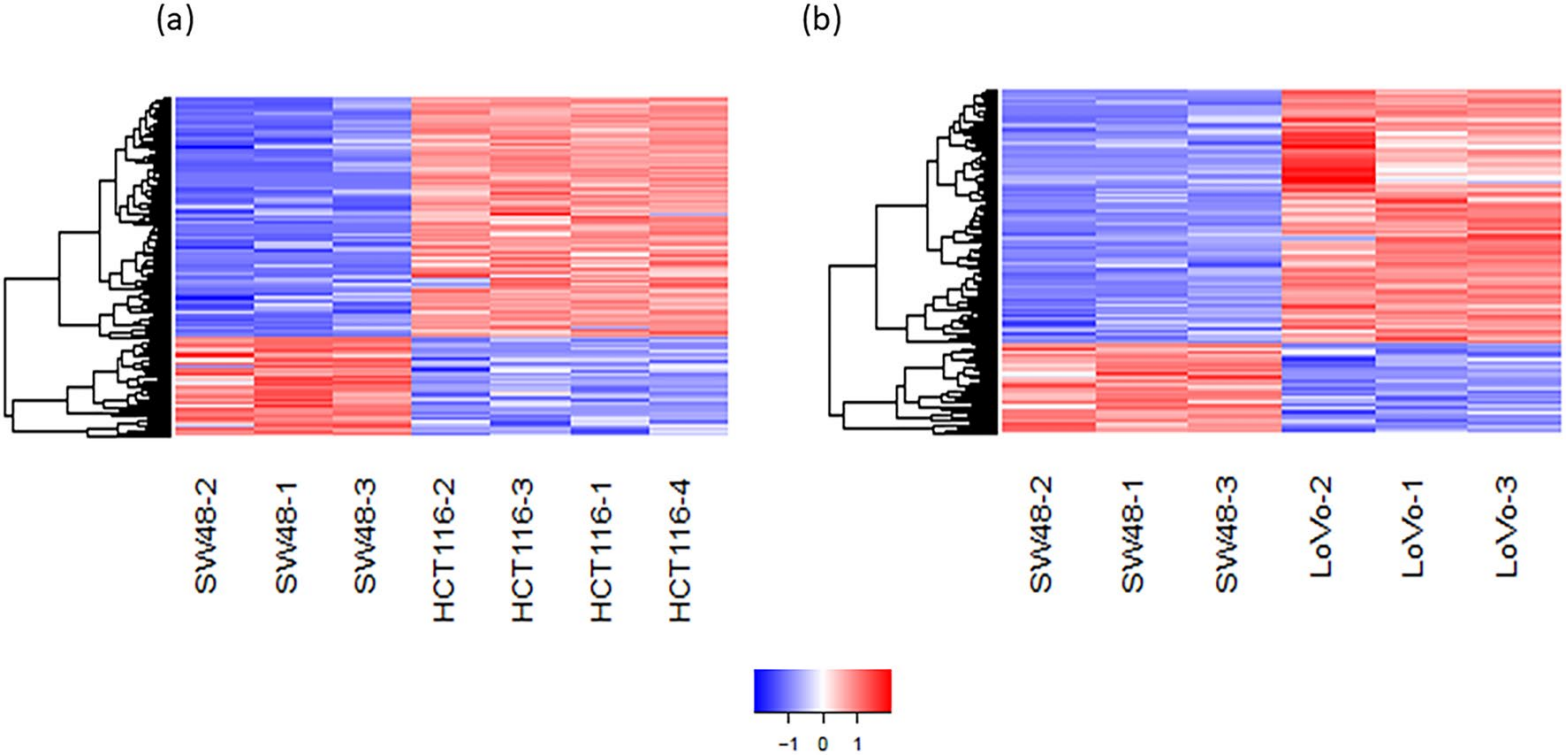
Heatmap plots based on the DEGs showing clustering of mtKRAS and wtKRAS cells lines into two distinct groups based on DEGs. (**a**) Clustering of HCT-116 and SW48 samples. (**b**) Clustering of LoVo and SW48 samples. Red shows overexpression and blue indicates underexpression with absolute log2FC > 1.5 and adjusted p-value < 0.05. Expression data are represented as normalized values (Z‑scores). Heatmap plot was created using gplots package in R environment and variance stabilizing transformation (VST) was applied to the normalized count data from DESeq2 package.

In this study, a multi-step strategy was developed to identify DEGs and differentially-expressed non-coding RNAs (DEncRNAs) (Fig. 1), allowing to distinguish the mechanisms involved in the KRAS mutation-induced malignancy by using publicly available datasets. In this regard, significant DEGs were first recognized by comparing the gene expression profiles between mtKRAS (HCT-116 and LoVo) and wtKRAS CRC cells (SW48). A total of 1979 and 1984 genes reached the criteria of 1.5-fold changes and adjusted p-value < 0.05 in HCT-116 versus SW48 and LoVo versus SW48, respectively (Supplementary Datas 1 and 2). Based on the results, 600 DEGs consisting of 492 coding and 108 non-coding (including 88 lncRNAS and miR-23b) Ensembl gene IDs were overlapped between the two comparisons. The identified DEGs with 423 up- and 149 downregulated gene IDs, in mtKRAS samples compared to wtKRAS, could be assigned to a KRAS-dependent gene expression signature (Supplementary Data 3). The cluster analysis of 600 DEGs revealed the significant differences between the wtKRAS and mtKRAS CRC cell lines (Fig. 2). Principle component analysis (PCA)-based multidimensional scaling visualization, linearly separated CRCs with and without KRAS mutation, on the basis of the gene expression data (Supplementary Data 4).

### Gene set enrichment analysis (GSEA)

**Figure 3:**
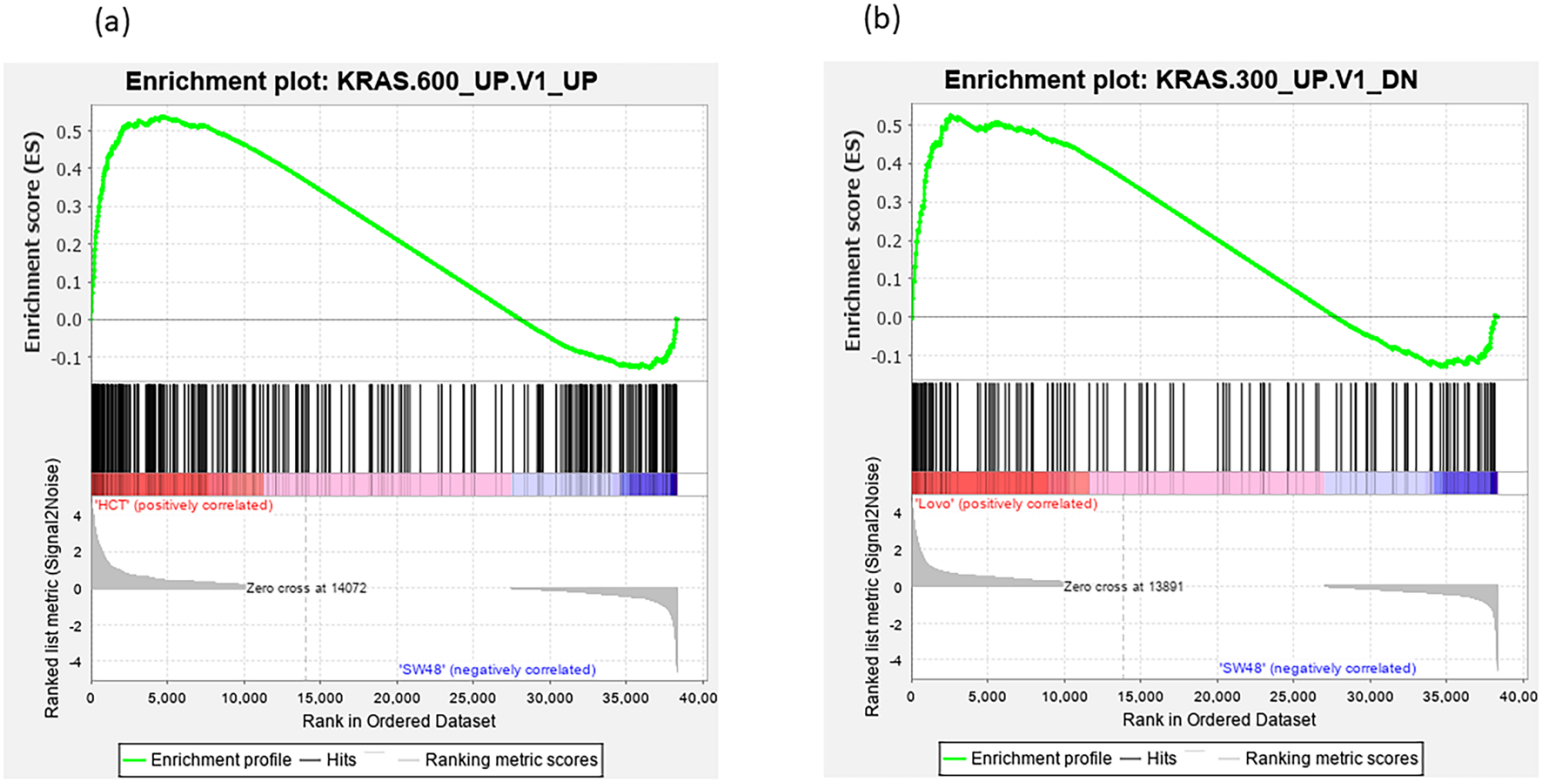
GSEA analysis for oncogenic signature gene sets. (**a**) Enrichment plot of GSEA analysis for HCT-116. (**b**) Enrichment plot of GSEA analysis for LoVo. Enrichment plot on the whole transcriptome of the CRC cell lines showing the up-regulation of KRAS signaling oncogenic signature in HCT-116 vs SW48 and LoVo vs SW48. FDR < 25% after 1000 random permutations was set as the cut-off criterion.

The results obtained from the GSEA analysis of the overrepresented KRAS signaling gene set reflected the clustering of LoVo and HCT-116 (mtKRAS) cells versus SW48 (wtKRAS) ones into two distinct groups (Supplementary Table 1). Additionally, GSEA was performed on the whole transcriptome of the CRC cell lines by using GSEA v2.07 software with 1000 gene set permutations to elucidate the discrimination of mtKRAS CRC cell line from wtKRAS one in the oncogene-associated signatures. Oncogenic signatures were derived from the Molecular Signatures Database v4.0. The results of GSEA indicated upregulated oncogenic signature gene sets like KRAS signaling in mtKRAS compared to the wtKRAS, while no significant oncogenic signature was upregulated while analyzing the use of SW48 versus mtKRAS cell lines as phenotype labels (Fig. 3).

### GO analysis and signaling pathway enrichment of DEGs

**Figure 4:**
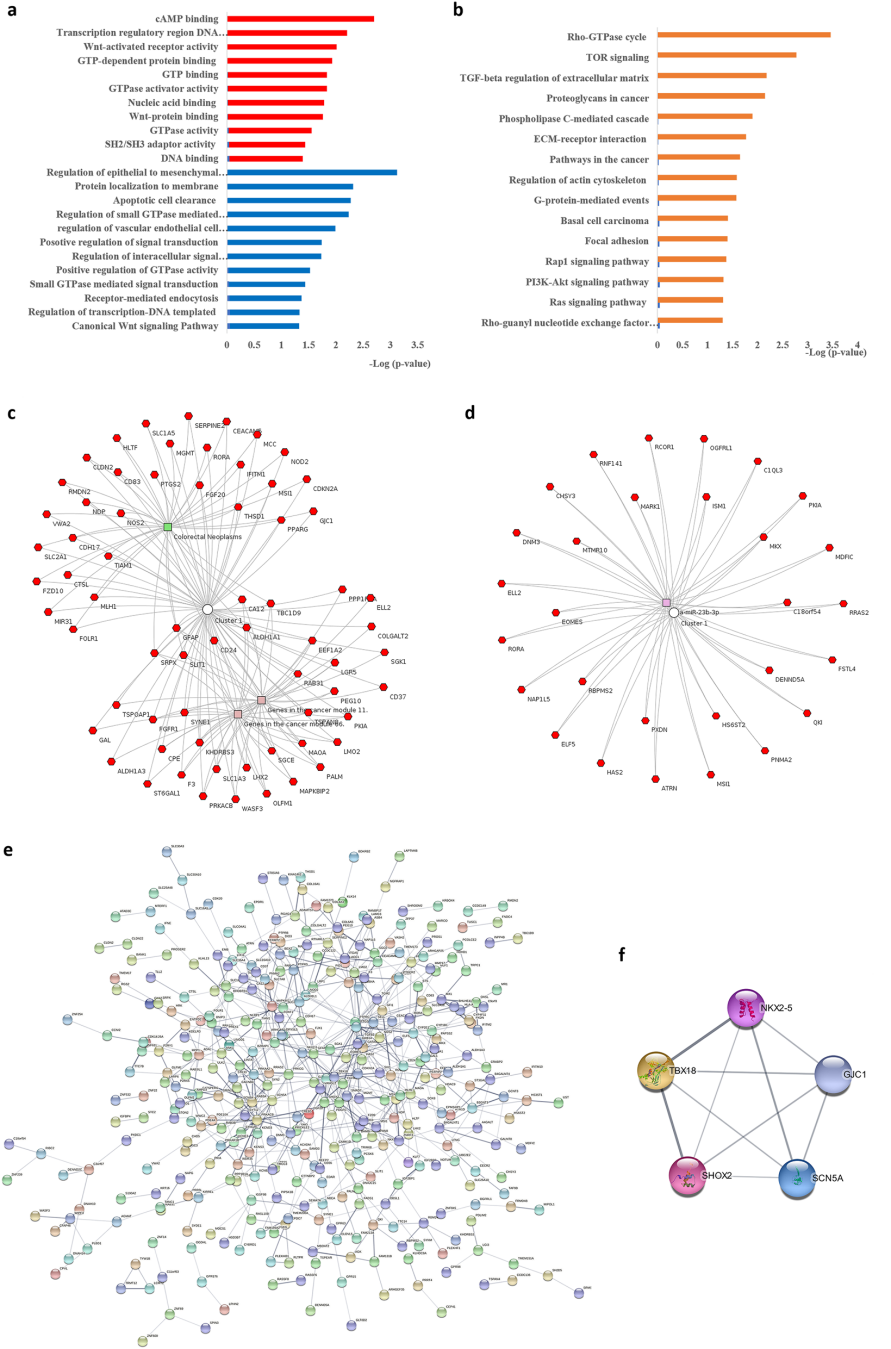
Functional enrichment analysis. (**a**) GO analysis of differentially expressed genes (DEGs) based on the molecular function (Red) and biological process (Blue). (**b**) KEGG pathway analysis of DEGs. (**c**) Functional enrichment analysis by Toppcluster. (**d**) Cluster of miR-23b and its target DEGs. (**e)** Protein–protein interaction network of DEGs. (**f**). One functional model identified from the PPI network by MCODE.

Figure 4 displays the results related to the functional enrichment analysis of the 600 DEGs. All DEGs were computationally uploaded to the DAVID and EnrichR resources for the better revelation of the molecular and cellular mechanisms underlying the carcinogenicity of KRAS mutations. Based on the results of GO enrichment analysis, the DEGs were divided into 23 functional categories including 11 molecular function terms and 12 biological processes (Fig. 4a). Regarding the biological process group, DEGs were enriched in regulation of epithelial to mesenchymal transition (EMT), regulation of small GTPase mediated signal transduction, regulation of vascular endothelial cell proliferation, and positive regulation of GTPase activity. In the molecular function category, DEGs were mainly enriched in the GTP-dependent protein binding, GTP binding, GTPase activator activity, GTPase activity, Wnt-protein binding. The results of KEGG pathway enrichment analysis demonstrated that DEGs were mainly enriched in Rho-GTPase cycle, TOR signaling, TGF-beta regulation of extracellular matrix, proteoglycans in cancer, pathways in the cancer, PI3K-Akt signaling pathway, and RAS signaling pathway (Fig. 4b).

Further, ToppCluster was used for implementing a multi-cluster gene functional enrichment analysis to obtain a functional modular map based on the human disease and computational features. The genes in the cancer module (11 and 86) and colorectal neoplasm were significantly enriched for computational and human disease genes, respectively (Fig. 4c). Furthermore, a significant number of the DEGs, known as miR-23b targets, was obtained by considering their feature enrichment analysis by using ToppCluster (with Bonferroni correction and a 0.05 p-value cut-off) (Fig. 4d).

### PPI network of DEGs

To verify the functional connectivity of the DEGs, the STRING database was interrogated to dissect the PPI networks. A total of 600 DEGs were analyzed by STRING database and following the removal of the partially-connected and isolated nodes, a network of 474 nodes with 601 edges was constructed by using the Cytoscape software (Fig. 4e). The top 10 hub genes with at least 10 degrees were PTPRC, PPARG, PRKACB, GATA3, PTGS2, CDKN2A, SCN5A, GFAP, CDH17, and EOMES, with the maximum node degree of which was observed in PTPRC as 25 (Fig. 4e). One functional module was identified from the PPI network of DEGs by using the MCODE composed of 5 nodes and 10 edges (Fig. 4f).

### Interaction between co-expressed DEncRNAs and DEGs

Among DEncRNAs, 12 differentially-expressed lncRNAs exhibited possible interactions with some DEGs (Table 1). The results related to the interaction analysis of co-expressed DEG-DElncRNA predicted the presence of several mechanisms for lncRNAs targeting mRNAs. The co-expression analysis revealed the DElncRNAs regulating the expression of the genes which are majorly enriched in actin cytoskeleton regulation, gap junction, and Rap1, RAS, PI3K-AKT, and MAPK signaling pathways. Thus, these potential pathways which are all recognized as the cancer-related pathways could be described in the context of the KRAS mutation.

**Table 1:** Interaction network of the co-expressed DElncRNAs and their target DEGs.

GENE-ID	LncRNA			mRNA		
	Gene symbol	Expression	GENE-ID	Gene symbol	Expression	Mechanism
ENSG00000261713	SSTR5-AS1	Up	ENSG00000162009	SSTR5	Up	Antisense to SSTR5
ENSG00000246695	RASSF8-AS1	Up	ENSG00000123094	RASSF8	Up	Antisense to RASSF8
ENSG00000225778	PROSER2-AS1	Up	ENSG00000148426	PROSER2	Up	Antisense to PROSER2
ENSG00000232295	AL589935.1	Up	ENSG00000119900	OGFRL1	Up	Antisense to OGFRL1, Targeting miR-23b
ENSG00000236548	RNF217-AS1	Up	ENSG00000146373	RNF217	Up	Antisense to RNF217
ENSG00000259953	AL138756.1	Up	ENSG00000106868	SUSD1	Up	Overlapping with SUSD1
ENSG00000267147	LINC01842	Up	ENSG00000120820	GLT8D2	Up	Targeting miR-4752
ENSG00000268191	AC010503.2	Up	ENSG00000104833	TUBB4A	Up	Antisense to TUBB4A
ENSG00000269825	AC022150.4	Up	ENSG00000167766	ZNF83	Up	Sense intronic to ZNF83
ENSG00000270504	AL391422.4	Up	ENSG00000168994	PXDC1	Up	Sense intronic to PXDC1
ENSG00000272159	AC087623.3	Up	ENSG00000077782	FGFR1	Up	Cis-targeting
ENSG00000237517	DGCR5	Down	ENSG00000123094	RASSF8	Up	Targeting miR-195
ENSG00000237517	DGCR5	Down	ENSG00000145431	PDGFC	Up	Targeting miR-195

Expression level shows the expression in mtKRAS versus wtKRAS.

### Transcription factor analysis

An EnrichR web-based tool was used for searching the overrepresented TFs regulating the identified DEGs. EnrichR was utilized for exploring the relevant TFs to find the possible regulatory mechanisms which may affect these target genes. The analysis was performed through detecting the binding motif sites in the gene list using the PWMs from TRANSFAC and JASPAR (Table 2). The regulatory biomolecules influencing the transcriptional regulation of DEGs were discovered by considering a statistical significance level of p-value < 0.01. Overall, 11 TFs of TFAP2A, SP1, ZBTB7A, HNRNPK, EGR1, TEAD4, HOXD9, SP3, PCBP1, MTF1, and UBTF were detected, which are mostly related to colon malignancy.

**Table 2:** List of TFs regulating the identified DEGs using Enrichr Tool through scanning the TRANSFAC and JASPAR databases.

Symbol	Description	Feature
TFAP2A	Transcription factor AP-2, alpha	Reduced expression in CRC progression
SP1	Specificity protein 1	High expression related to poor prognosis of CRC
ZBTB7A	Zinc finger and BTB domain-containing 7A	As an oncogene in CRC
HNRNPK	Heterogeneous nuclear ribonucleoprotein K	Overexpression with poor prognosis in CRC
EGR1	Early growth response-1	CRC cell proliferation
TEAD4	TEA domain family member 4	Promotion of CRC tumorigenesis
HOXD9	Homeobox Protein Hox-D9	Promotion of metastasis in CRC
SP3	Specificity protein 3	Induction of EMT of CRC
PCBP1	Poly(RC) Binding Protein 1	Induction of drug resistant CRC
MTF1	Metal Regulatory Transcription Factor 1	Association with EMT in CRC
UBTF	Upstream Binding Transcription Factor	Promotion of CRC cell proliferation

### In-silico verification of hub genes and TFs

**Figure 5:**
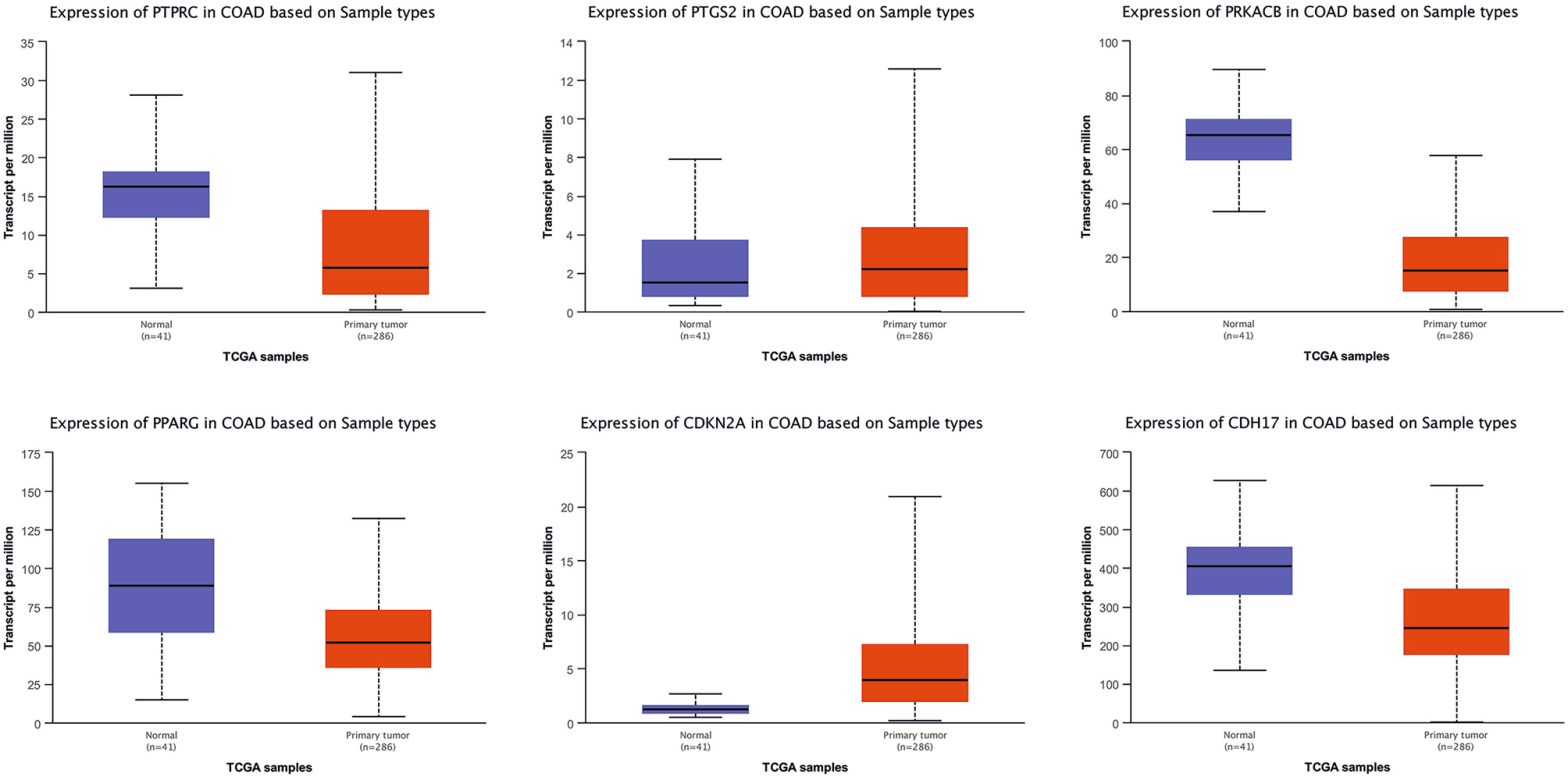
The box-plots show the expression of some of the hub genes with statistically significant differential expression in two normal and tumor groups.

**Figure 6:**
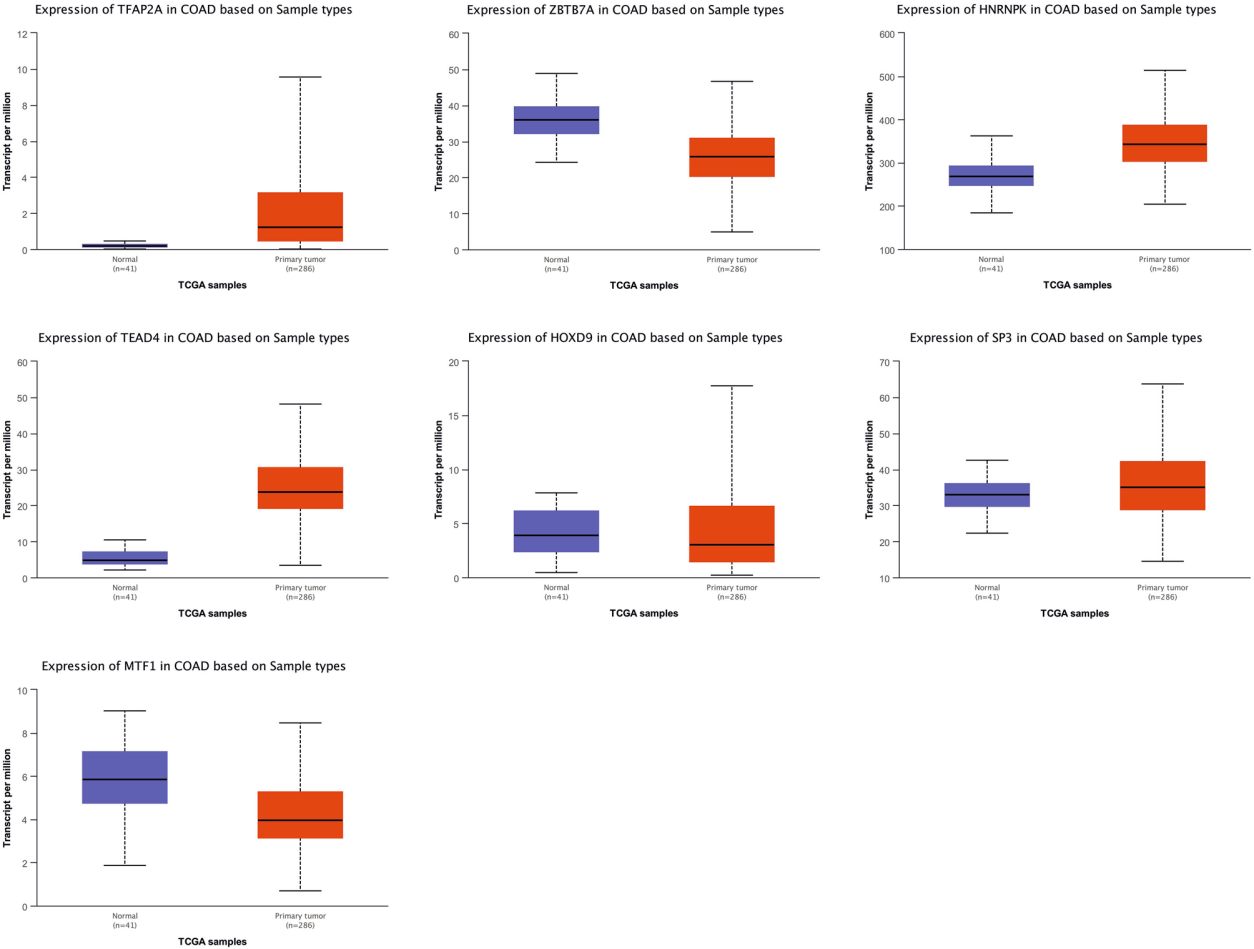
The box-plots show the expression of some of the TFs with statistically significant differential expression in two normal and tumor groups.

In order to test the stability of the results, the expression levels of hub genes and TFs were further explored in the same CRC cell lines using from Gene Expression Atlas database for verification (Table 3). In general, the expression levels related to 77% of these genes are in line with the results of differential expression analysis, which reflects the reliability and reproducibility of the results of the present study. In addition, the expression level of hub genes and TFs was compared with the expression data of CRC tissue samples deposited in TCGA databank. Regarding the simulations, 60% of hub proteins and 70% of reporter TFs were considered as distinct biomarker sets exhibiting their contribution to the discrimination of normal and tumor patient samples (Figs. 5 and 6).

**Table 3:** Validation of the expression level of hub gene in The Gene Expression Atlas.

Hub gene	Bioinformatics results				Verification results		
	Log2FC		Expression status		TPM		
	SW48 vs LoVo	SW48 vs HCT-116	SW48 vs LoVo	SW48 vs HCT-116	HCT-116	LoVo	SW48
PPARG	− 7.81	− 4.63	Down	Down	22	98	3
PTGS2	− 9.44	− 5.72	Down	Down	0/6	187	0
PRKACB	− 8.30	− 10.43	Down	Down	164	24	9
GATA3	− 7.44	− 6.28	Down	Down	4	5	0
CDKN2A	− 3.67	− 6.46	Down	Down	11	4	13
PTPRC	5.72	7.39	Up	Up	No evidence	0	2
SCN5A	− 6.54	− 7.43	Down	Down	5	5	6
GFAP	− 8.80	− 6.64	Down	Down	No evidence	No evidence	No evidence
EOMES	− 5.72	− 6.54	Down	Down	3	1	0

### Survival Analysis of hub genes and transcription factors

**Figure 7:**
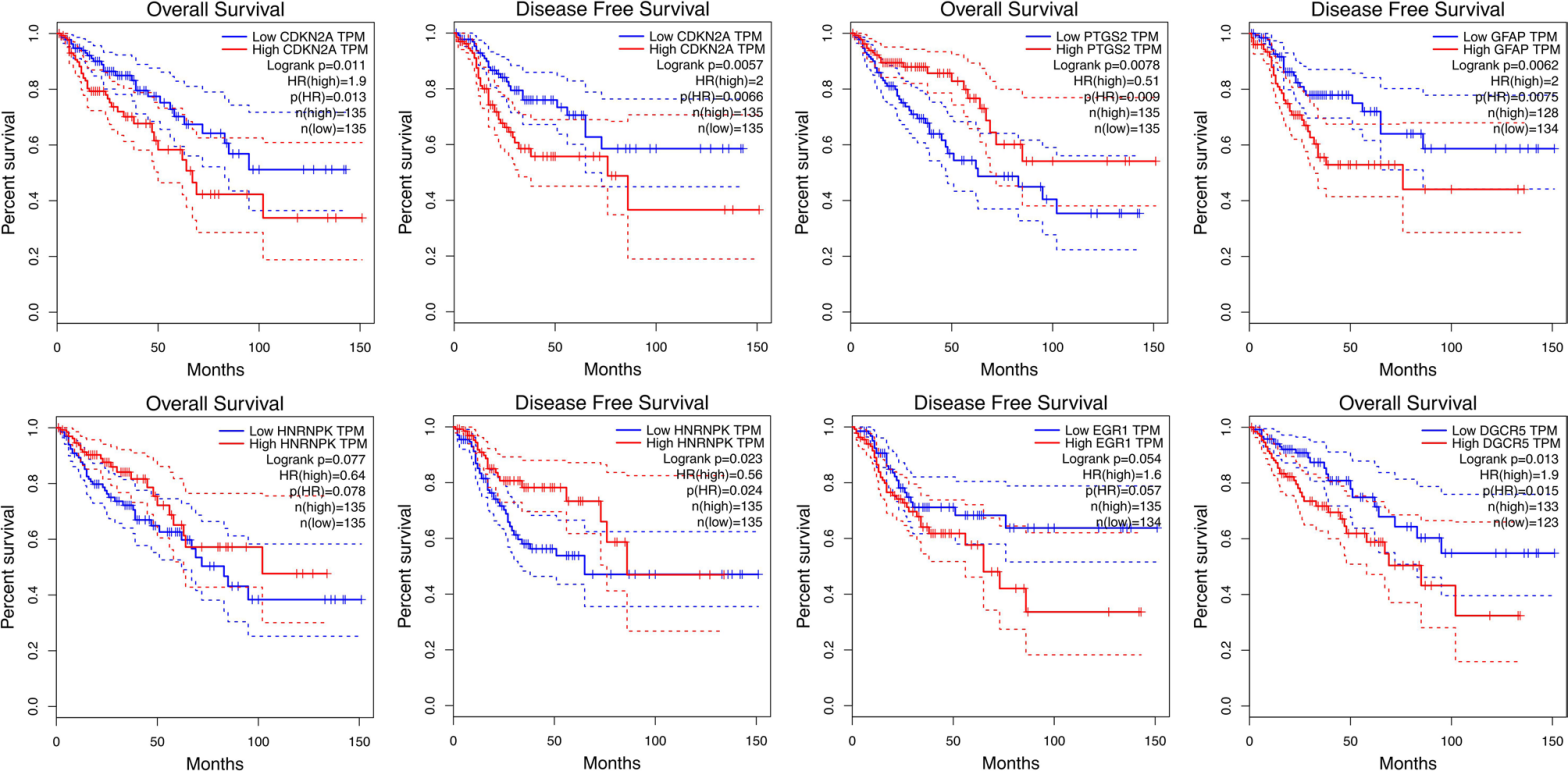
The survival analysis of the hub genes, TFs, and lncRNAs in the prognosis of colorectal cancer. The Kaplan–Meier plot indicates the prognostic ability of hub genes, TFs, and lncRNAs signatures in CRC.

The Kaplan–Meier survival analyses of the hub genes, TFs, and lncRNAs in CRC patients was evaluated using GEPIA tools (Supplementary Figs. S1–S6). Among the hub genes, PTGS2 and CDKN2A, and GFAP were associated statistically significant with overall survival and disease-free survival of CRC patients. In addition, among the TFs, HNRNPK and EGR1 were associated statistically significant with overall survival and disease-free survival, respectively. Survival analysis for lncRNAs which were included in the interaction network with DEGs (Table 1) showed the significant connection between DGCR5 and overall survival of CRC patients (significance level at log-rank p < 0.05) (Fig. 7).

### Quantitative real-time PCR verification

**Figure 8:**
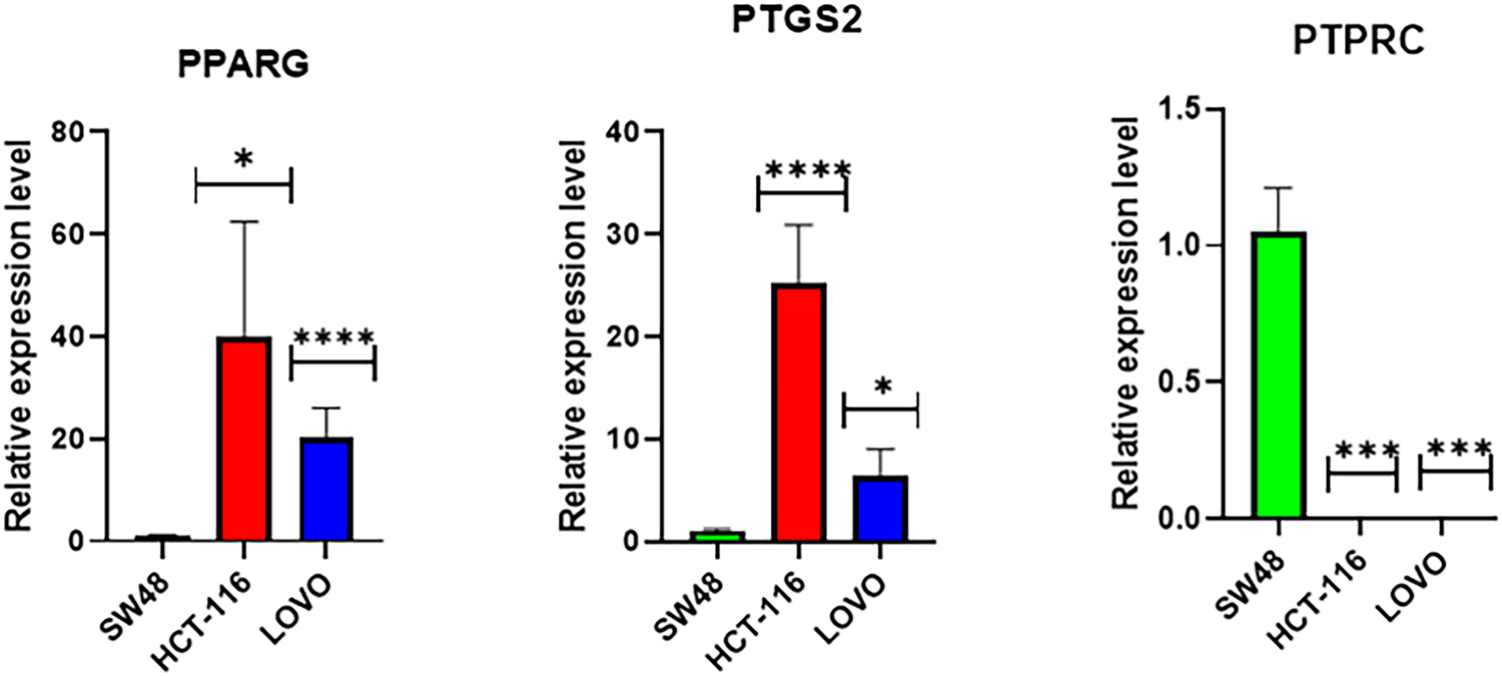
Quantitative real-time PCR validated three dysregulated genes in association with KRAS mutation. Expressions of PPARG and PTGS2 were significantly elevated in KRAS-mutant cell lines (HCT-116 and LoVo) compared non-mutant cell line (SW48). The expressions of PTPRC in SW48 compared with other cell lines was upregulated.

To validate the reliability of RNA-seq results, the expression level of the three selected hub genes possessing the highest degrees was measured using qRT-PCR between KRAS mutant CRC cell lines and their corresponding wtKRAS one (Fig. 8). Based on the results, PPARG and PTGS2 transcripts were significantly upregulated in KRAS mutant cell lines compared to the wtKRAS. In contrast, an elevation was obtained for the expression level of PTPRC in SW48 in comparison with the LoVo and HCT-116. The results are consistent with those of RNA sequencing, which confirms the reliability of RNA sequencing analysis.

## Discussion

The recent advances in high-throughput technologies such as next-generation sequencing have enabled the global analyses of the key genes, lncRNAs, and miRs playing important roles in the CRC malignant transformation. In this study, the transcriptional profiles of mtKRAS and wtKRAS CRC cell lines were analyzed and compared by considering publicly available RNA-seq data to identify the critical signaling pathways involved in the tumorigenesis of the KRAS-driven CRCs.

Regarding the bioinformatics analysis of KRAS mutation effects on carcinogenesis, the RNA-seq data sets were selected from the largest publicly available repository of high-throughput sequencing data. Further, 600 DEGs were detected in comparing the transcriptome of mtKRAS cell lines with the wtKRAS one. The results of GO term analysis indicated the enrichment of DEGs in the biological processes such as the regulation of EMT, regulation of small GTPase mediated signal transduction, regulation of vascular endothelial cell proliferation, and positive regulation of GTPase activity, which are the critical causes of malignant transformation and cancer progression^22,23^. Epithelial plasticity or EMT is a key cellular program that can be activated by KRAS contributing to tumor progression^24^. Rho GTPases, the members of the Ras superfamily of small GTPases, are known as important signal transducers in the signaling pathways regulating cell proliferation, migration, survival, and death, all of which are the cellular processes crucial for cancer progression^22^.

The functional enrichment analysis revealed that DEGs were mainly enriched in key pathways like Rho-GTPase cycle, TOR signaling, TGF-beta regulation of extracellular matrix, proteoglycans in cancer, pathways in the cancer, PI3K-Akt signaling pathway, and RAS signaling pathway, which are all cancer-related pathways could be involved in KRAS-associated carcinogenesis^25–27^. Another functional enrichment analysis of the putative DEGs associated with KRAS was generated from ToppCluster. The results of analyzing based on the human disease and computational features were corresponded to the genes of cancer module and colorectal neoplasms, respectively. Therefore, the signaling pathways attributed to the DEGs under study are mainly involved in cancer and may provide new insights for understanding the cellular events underlying KRAS tumorigenesis.

In addition, 10 hub genes were identified from the constructed PPI network in terms of the highest degree, which included PTPRC, PPARG, PRKACB, GATA3, PTGS2, CDKN2A, SCN5A, GFAP, CDH17, and EOMES. Peroxisome proliferator activated receptors (PPARs) are the nuclear hormone receptors contributing to the genetic control of many cellular events. It, as a critical TF, regulates the important cellular functions which contribute to the regulation of metabolism and inflammation, and promotion of tumor survival and growth^28^. Some researchers have found the possible contribution of the crosstalk between PPARG signaling and epigenetic machinery to CRC development^29^. PPARG is overexpressed in different human cancers such as CRCs and pancreatic one, where its upregulation is correlated with increased pathological grade^30–32^. Further, prostaglandin E2 enhances PPARG transcriptional activity to promote CRC cell survival in vitro and intestinal tumorigenesis in mice^33^. JAK/STAT signal transduction pathway plays an essential role in the CRC malignancy through inactivating tumor suppressor genes and activating oncogenes[3435]. Receptor-type protein tyrosine phosphatases (PTPRs) are considered as a subgroup of protein tyrosine phosphatases with a crucial role in the cellular signaling pathways related to proliferation, survival, apoptosis, migration, and invasion. The genetic and epigenetic alterations of the phosphatases lead to aberrant cell signaling, which suggests that the PTPRs are critical components in the carcinogenesis of several cancers such as CRCs^36,37^. Consistent with previous studies, protein kinase CAMP-activated catalytic subunit beta (PRKACB) had a significant discrepancy of expression in normal and tumor tissues and it was also associated with poor patient-free survival and overall survival^38^.

Numerous studies have linked CRC to inflammation and prostaglandin-endoperoxide synthase (PTGS) 2 expression^39^. Other epidemiological, clinical, and animal studies have reported PTGS2 and epithelial growth factor (EGF) signaling pathways with key roles in promoting CRC growth and metastasis^40^. GATA3, as another hub gene in the present study, is expressed in CRC with a suppressive effect on the invasive behavior of CRC cells^41^. Methylation of cyclin dependent kinase inhibitor 2A (CDKN2A) promoter and subsequent gene silencing have been documented in many tumors including colon cancer and it has been associated with the CpG island methylator phenotype^42^. Microarray data established a definite role of sodium voltage-gated channel alpha subunit (SCN5A) as a high level regulator of CRC invasion network, including genes involved in Wnt signaling, cell migration, and cell cycle control^43^. The reduction in the density of glial fibrillary acidic protein (GFAP) glial cell type in the enteric nervous system was related with tumor grading of CRC and the inverse variation with the tumor proliferative activity and tumor-infiltrating leukocytes which could serve as a potential prognostic factor in this cancer^44^. Interaction between Cadherin-17 (CDH17) and α2β1 integrin has been revealed to regulate cell proliferation and adhesion in CRC cells causing liver metastasis^45^. In patients with CRC, high expression of Eomesodermin (EOMES) was associated with poor overall survival compared with individuals exhibiting low EOMES levels. However, a divergent role in cancer development, with tumor suppressor or oncogenic activities, has been reported for EOMES depending on stage and tissue context^46^.

Among the hub genes, the the three selected genes with the significant role in CRC tumorigenesis were selected for further verification by qPCR. The expression of PPARG, PTGS2, and PTPRC was validated in the CRC cell lines with and without KRAS mutation. The results represented the higher expression of PPARG and PTGS2 in KRAS mutant cell line than the SW48 (wtKRAS), which is in agreement with SRA data. The PTPRC expression was significantly upregulated in SW48 compared with HCT-116 and LoVo as the KRAS mutant samples.

To date, an increasing number of dysregulated lncRNAs with critical roles in human cancers such as CRC has been recognized^47^. In the present study, the dysregulated lncRNAs between mtKRAS and wtKRAS CRC cell lines were analyzed by using RNA-seq datasets from SRA. Among the lncRNAs, some with no annotation were excluded, and then the effects of the lncRNAs were highlighted, which could be included in the co-expressed DEG-DElncRNAs interaction network. The results of the pathway analysis of DEGs, which could potentially interact with DElncRNAs, reflected their major involvement in the actin cytoskeleton regulation, and Rap1, Ras, PI3K-AKT, and MAPK signaling pathway, and a positive correlation with cancer hallmarks. Various mechanisms are available for lncRNAs to activate, repress, or modulate the expression of target genes^48^. Cis-acting lncRNAs, which constitute a substantial fraction of lncRNAs, regulate the expression of their target genes in a location-dependent manner^49^. They can repress or activate their target genes through different mechanisms according to the possible architectures of lncRNA-target units. Antisense lncRNAs overlap their target genes in the antisense orientation^50^, while the sense ones overlap their target genes in the sense orientation and are typically contained within target gene introns^51^. Based on the competing endogenous RNA (ceRNA) hypothesis, the sequestration of the miRs from their mRNA target is another mechanism for regulating gene expression by lncRNAs^52–54^. Regarding the present study, the differential expression analysis of non-coding transcripts revealed the significant upregulation of miR-23b in wtKRAS cell line compared to the mtKRAS one. The downregulation of miR-23b in plasma is associated with poor prognosis among CRC patients^55^. Additionally, the PDGFR-modulated miR-23b cluster suppresses lung tumorigenesis by targeting the multiple members of KRAS and NF-kB pathways^56^. The results obtained from the feature enrichment analysis of DEGs with ToppCluster represented a significant number of DEGs as miR-23b targets. In the present study, a lncRNA-miRNA-mRNA cross-talk was recognized among AL589935.1-miR-23b-OGFRL1. Further, miR-23b was downregulated while upregulating AL589935.1 and OGFRL1 in mtKRAS versus wtKRAS, by indicating the ceRNA hypothesis. Considering some studies proposing the potential role of opioid signaling axis in cancer, the results can be utilized for understanding the molecular basis of the KRAS mutation-induced tumorigenesis^57^.

The significant TFs regulating the DEGs have been discovered, which play a role in colon malignancy. Among TFs, TFAP2A expression reduces in high-grade colorectal adenocarcinomas^58^, the high expression of SP1 is ascribed to CRC poor prognosis^59^, and ZBTB7A is known as an oncogene in CRC^60^. Furthermore, POU2F1 regulates colon malignancy^61^, the overexpression of HNRNPK is attributed to CRC poor prognosis^62^, EGR1 is related to CRC cell prolifration^63^, and TEAD4 and HOXD9 promote CRC tumorigenesis^64,65^. The survival analysis of the hub genes and TFs reflected their high potential as prognostic biomarkers with worse survival outcomes of the CRC patients.

In the present study, the dysregulated genes tending to be differentially expressed in the context of KRAS mutation were examined based on the SRA database, which included the hub genes derived from the PPI network such as PPARG, PTGS2, PTPRC, CDKN2A, and PRKACB. In addition, DElncRNAs were identified and the putative molecular interactions between DEGs and lncRNAs was provided. The results obtained from the GO and pathway analysis of dysregulated genes and lncRNAs proposed their role in the cancer-related pathways. Further, 11 overrepresented TFs regulating the identified DEGs were found, which were mostly related to colon malignancy. The survival analysis of hub genes and TFs revealed the contribution of almost all genes and TFs in discriminating the risk groups. The results of analyzing the differential expression of the non-coding transcripts represented that miR-23b was significantly upregulated in wtKRAS. Furthermore, a lncRNA-miRNA-mRNA crosstalk was observed among AL589935.1-miR-23b-OGFRL1. AL589935.1 and OGFRL1 were upregulated, and miR-23b was downregulated in mtKRAS versus wtKRAS, which suggests the possible ceRNA effect. The altered expression of PPARG, PTGS2, and PTPRC in mtKRAS and wtKRAS cells was confirmed by using qPCR and *in-silico* verification using Gene Expression Atlas and TCGA databases.

Some limitations of this study should be considered for the better interpretation of the results. The results were based on the transcriptional profile of cancer cell lines, which should be verified by patient sample data. However, the results were confirmed by the *in-silico* validation using TCGA database. Finally, more molecular biology experiments and computational method analysis for big data are required to clarify the relationship among the predicted regulatory lncRNAs, miR-23b, and DEGs.

## Conclusion

In the present study, a comprehensive bioinformatics analysis of DEGs was performed, which may be involved in the KRAS-driven tumorigenesis. Along with affecting the KRAS-associated pathways, the possible effect of KRAS mutation on the gene expression of other signaling pathways such as those related to Rho-GTPase cycle, TOR signaling, TGF-beta regulation of extracellular matrix, proteoglycans in cancer, and pathways in the cancer is one of the important results of this study. The altered pathways may be associated with more significant cancer hallmarks induced by KRAS oncogenic effects. The identification of the differentially-expressed lncRNAs with crucial roles in regulating the potential cancer-related pathways may provide reference lncRNAs for diagnosing and treating KRAS mutant CRC. Additionally, a set of hub genes was recognized in the constructed PPI network, which may be considered as therapeutic targets. These discriminating genes such as PPARG, PTPRC, PTGS2, CDKN2A and PRKACB were reported with regard to their role in colorectal carcinogenesis although their expression was not described in the context of KRAS status in CRCs. Finally, further experiments are needed for understanding the corresponding roles and molecular mechanisms of the lncRNAs and mRNAs in detail to specify the mechanism of the KRAS-mediated tumorigenesis in CRC.

## Materials and methods

### Data collection

The RNA-seq data were extracted from the Sequence Read Archive database (https://www.ncbi.nlm.nih.gov/sra), which corresponded to the three human CRC cell lines of HCT-116 (SRR1030462, SRR1030463, SRR1756569, and SRR8615282), LoVo (SRR1756570, SRR8532655, and SRR8616185), and SW48 (ERR208907, SRR3228439, and SRR8615504). HCT-116, LoVo, and SW48 cell lines were obtained from The Research Institute of Biotechnology, Ferdowsi University of Mashhad.

### Data preprocessing and differential expression analysis

The data were downloaded as SRA files and converted to FASTQ format using fastq-dump from the SRA toolkit (https://github.com/ncbi/sra-tools). The RNA-seq files were filtered for quality by using FLEXBAR, AfterQC, and Trimmomatic^66–68^. In addition, the human genome (http://ftp.ensembl.org/pub/release95/fasta/homo_sapiens/dna/Homo_sapiens.GRCh38.dna.toplevel.fa.gz) was indexed using Bowtie2^69,70^. The filtered reads were mapped with Bowtie2, which the resultant SAM files were processed by using the htseq-count program for counting the aligned reads overlapped with the exons of each gene, given the GTF file (https://ftp.ensembl.org/pub/release95/gtf/homo_sapiens/Homo_sapiens.GRCh38.98.gtf.gz)^71^. Further, the expression profiles of HCT-116 and LoVo cell lines (mtKRAS) were compared with the transcriptome profile of SW48 one (wtKRAS) (as the control). The read counts were normalized for all samples and differentially-expressed Ensembl gene IDs were identified by using the DESeq2 package (version 1.38.0) from Bioconductor in R environment (version 3.6.1, https://www.rproject.org/)^72^. Furthermore, an adjusted p-value < 0.05 and a |log2FC|≥ 1.5 were defined as screening criteria. The Ensembl gene IDs with differential expression were annotated with Ensembl Biomart (https://asia.ensembl.org/biomart/martview). The Ensembl and human genes (GRCh38.p13) (as mart databases), as well as transcript type (as an attribute), were utilized for annotation to categorize the differentially-expressed Ensembl gene IDs into coding genes and non-coding RNAs. Hierarchical cluster analysis was performed to distinguish the differential expression of the overlapped DEGs in CRC samples with KRAS mutation compared to the control sample. Heatmap plot was created using gplots package in R environment and variance stabilizing transformation (VST) was applied to the normalized count data from DESeq2 package. Distance measurement and linkage analysis were based on Euclidean distance and complete linkage, respectively. Adjusted P value < 0.01 and a |log2FC|> 2 were defined as the cut-off criteria. Expression data are represented as normalized values (Z‑scores). Variations between CRC cell lines with and without KRAS mutations were also compared by PCA based on the multigene expression data.

### Gene set enrichment analysis

Gene set enrichment analysis (https://www.gsea-msigdb.org/) was applied on the whole transcriptome to explore the potential altered pathways of mtKRAS cell lines in comparison with those of the wtKRAS one. To identify significantly enriched gene sets associated with cancer, between two biological states related to the KRAS mutation, gene sets were obtained from C6-Oncogenic signatures database. Two gene lists were analyzed consisting of the total number of the DEGs in HCT-116 versus SW48 and LoVo versus SW48 with 1972 and 1984 genes, respectively. The new GSEA Ensemble CHIP files provide this analysis for RNA-seq data and in this study Human-ENSEMBLE-Gene-MSigDB chip was selected as the chip platform. False discovery rate (FDR) less than 25% after 1000 random permutations was set as the cut-off criterion.

### GO and pathway enrichment analysis of DEGs

To better understand the biological functions and potential mechanisms of overlapped DEGs in the mechanism of the KRAS-related tumorigenesis, we applied GO enrichment and pathway analyses. Briefly, GO analyses (www. geneontology.org) consisted of two components: biological process, and molecular function. Gene ontology analysis was performed by using the Database for Annotation, Visualization, and Integrated Discovery (DAVID) bioinformatics tool (version 6.8) (https://david.ncifcrf.gov/) and comprehensive gene set enrichment analysis web server EnrichR (https://amp.pharm.mssm.edu/Enrichr)^73,74^. Pathway analyses was carried to investigate the potential significant KEGG pathways using DAVID database and EnrichR resource^75,76^. The GO terms and KEGG pathways with p-value < 0.05 were considered as significantly enriched function annotations. Additionally, functional enrichment analysis was carried out by ToppCluster (https: //toppcluster.cchmc.org) as the other bioinformatics resource for detecting the functional enrichment of the candidate genes^77^. The ToppCluster output with Bonferroni correction and 0.05 p-value cut-off was visualized in Cytoscape software (version 3.7.2) (https://cytoscape.org/)^78^.

### Protein–protein interaction network and module analysis

The potential DEG-encoded protein interactions were explored by using the Search Tool for the Retrieval of Interacting Genes (STRING, version 11.0) (https://string-db.org)^79^. The PPI network was visualized using Cytoscape 3.9.0 software, which the PPI confidence score lower than 0.4 and genes in the network with at least 10 degrees were respectively set as significant and hub genes. Subsequently, the Cytoscape plugin Molecular Complex Detection (MCODE) (version 2.0.0) was utilized to screen the modules of the PPI network with scores > 3, node ≥ counts 5, node score = 0.2, k-core = 2, and maximum depth = 100 as cut-off criteria.

### Interaction analysis between co-expressed DEncRNAs and DEGs

According to the results of differential expression analysis, DElncRNAs were assessed for their co-expression with their mRNA targets. Finding the relevant co-expressed DEG-lncRNAs was based on the two different strategies. The first approach was on the basis of the hypothesis posing the ability of lncRNAs and mRNAs to co-regulate each other by sharing common miRNA response elements competitively, and the cis-regulation of mRNAs with DElncRNAs was considered as the second solution. lncRNAs, as the key regulators of gene expression are likely to function as competing endogenous RNAs (ceRNAs) through miRNA sponging, thereby indirectly regulating the expression of the genes targeted by these miRNAs. With this concept, miRNA targets of lncRNAs were predicted by LncRNA2Target v2.0 (http://www.lncrna2target.org) and LncTarD (http://bio-bigdata.hrbmu.edu.cn/LncTarD)^80,81^. Then, miRTarBase (http://mirtarbase.mbc.nctu.edu.tw/) and DIANA-TarBase v8 (http://www.microrna.gr/tarbase) were employed for predicting mRNA targets of candidate miRNAs to find our DEGs among the gene targets^82,83^. Regarding cis-regulation analysis, the genes transcribed within a 300-kb window upstream or downstream of lncRNAs were considered as cis target genes according to the Ensembl genome browser. These cis-regulated genes by DElncRNAs were checked to find the common genes with our DEGs which shows the interaction between co-expressed DElncRNAs and DEGs.

### Transcription factor analysis

To identify overrepresented TF molecules from DEGs, TF discovery module was implemented, and processing was performed against both the TRANSFAC and JASPAR databases using EnrichR tool^74,84^. Based on the 35 different gene-set libraries, EnrichR computed enrichment and binding motif sites in the intended gene were detected through applying the position weight matrix (PWM) analysis from TRANSFAC and JASPAR^85^. The PWMs from TRANSFAC and JASPAR were utilized to scan the promoters of all human genes in the region of − 2000- + 500 from the transcription factor start site (TSS). Supposing the statistical significance of p-value < 0.05, TFs were recognized as reporter regulatory molecules, around which significant changes occur at the transcriptional level of DEGs.

### Evaluation of the prognostic performance of hub genes and TFs

The prognostic power of hub genes and TF regulatory biomolecules were analyzed through utilizing interactive web-based tool GEPIA (Gene Expression Profiling Interactive Analysis), which is based on the gene expression RNA-seq datasets of TCGA (The Cancer Genome Atlas) and GTEx (Genotype-Tissue Expression)^86^. This resource aids in in-silico validation of target genes and for identifying tumor sub-group specific candidate biomarkers, on the basis of the expression analysis of the query genes across normal and tumor samples. The statistical significance of differences in gene expression levels between the normal and tumor groups were summarized for hub genes, TFs, and lncRNAs.

### Culture of CRC cell lines

The human colon cancer cell lines HCT-116 and LoVo harboring KRAS mutation were selected as the mtKRAS ones. In addition, the SW48 cell line having wild-type *KRAS*, *BRAF*, *PIK3CA*, and *TP53* was adopted as the control in the RNA-seq comparisons with the other mtKRAS cell lines. LoVo and SW48 cells were grown at 37 °C under 5% CO_2_ in the RPMI medium (Invitrogen) supplemented with 10% FBS (Invitrogen) and penicillin (100 U/mL)-streptomycin (100 μg/mL) (Betacell). However, HCT-116 cells were grown at 37 °C under 5% CO_2_ in the DMEM media (Gibco) complemented with 10% FBS and penicillin–streptomycin.

### RNA extraction and quality monitoring

Total RNA was extracted from three independent experiments with a total RNA extraction kit (Parstous) according to the manufacturer's instructions. Further, RNA quantity and quality were measured on a Thermo Fisher Scientific NanoDrop 2000c spectrophotometer and an agarose gel electrophoresis. DNase treatment was performed by using a DNA-free DNase kit (SINACLON) and following the manufacturer's instruction.

### Quantitative real-time PCR (RT-PCR) verification

The qPCRs were conducted by using SYBR green dye (Parstous) in a thermal cycler (BioRad CFX96). Briefly, cDNA was reverse-transcribed from 1 μg RNA with random hexamers, oligo d(t) primers, and H-minus MMLV reverse transcriptase by using an Easy cDNA synthesis kit (Parstous). The qPCR reaction components included 2 μL of cDNA, 10 μL of SYBR green master mix, 1 μL of each primer (100 pmol/μL), and 6 μL of nuclease-free water. Thermal cycling program was pre-denaturation at 95 °C for 4 min, followed by 40 cycles of denaturation at 95 °C for 30 s, annealing at 60 °C for 30 s, and extension at 72 °C for 30 s. All experiments were performed in technical and biological triplicate, the results of which were normalized against the geometrical mean of the expression levels of two reference genes ACTB and GAPDH. Furthermore, the first three hub genes with the highest degree (PPARG, PTGS2, and PTPRC) were selected for qPCR assay. Table 4 summarizes primer sequences. The relative gene expression levels between samples were calculated through employing 2^−△△Ct^ method^87^.

**Table 4:** Gene names, primer sequences, amplicon sizes, and accession numbers of analyzed genes.

Gene	Primers	Amplicon size (bp)	Accession number
PPARG	F: TGGATGTCTCATAATGCCATCAGGT R: CTTTGGTCAGCGGGAAGGA	171	NM_001354666.3
PTGS2	F: CCTCAGACAGCAAAGCCTACC R: CGGTTTTGACATGGGTGGGA	135	NM_000963.4
PTPRC	F: AGTGGTTTGTTCTTAGGGTAACAGA R: ATGCCAAGAGTTTAAGCCACAAATA	142	NM_001267798.2
ACTB	F: TGGCACCACACCTTCTACAATGAG R: CAGCCTGGATAGCAACGTACA	160	NM_001101.5
GAPDH	F: GAAGGCTGGGGCTCATTTG R: GCTGATGATC TTGAGGCTGTTGT	127	NM_001256799.3

*PPARG* peroxisome proliferator activated receptor gamma, *PTGS2* prostaglandin-endoperoxide synthase 2, *PTPRC* Protein tyrosine phosphatase receptor type C, *ACTB* actin beta, *GAPDH* glyceraldehyde-3-phosphate dehydrogenase, *F* forward, *R* reverse, *bp* base pair.

### In-silico validation of the expression levels of hub genes

The expression levels of hub genes were validated in the Expression Atlas database (https://www.ebi.ac.uk/gxa/) providing information on gene expression patterns from RNA-seq, microarray studies, and protein expression in order to confirm the validity of the results^88^. The baseline expression of each hub gene was compared among three CRC cell lines by considering cancer cell line as the biological condition and transcripts per million (TPM) as data unit to represent expression level.

### Statistical analysis

All of the data were presented as the mean ± SD of three independent experiments (Prism-GraphPad Software) and analyzed through using unpaired Student's *t*-test (two-tailed). The p-values less than 0.05 were considered as statistically significant.

## Supplementary Information

Supplementary Figure S1.

Supplementary Figure S2.

Supplementary Figure S3.

Supplementary Figure S4.

Supplementary Figure S5.

Supplementary Figure S6.

Supplementary Legends.

Supplementary Data 1.

Supplementary Data 2.

Supplementary Data 3.

Supplementary Data 4.

Supplementary Table 1.
