# Supplementary figures and images for: Differential expression analysis of genes and long non-coding RNAs associated with KRAS mutation in colorectal cancer cells
Source: Sci Rep. 2022 May 13;12:7965. doi: 10.1038/s41598-022-11697-5 (PMC9106686; doi:10.1038/s41598-022-11697-5)

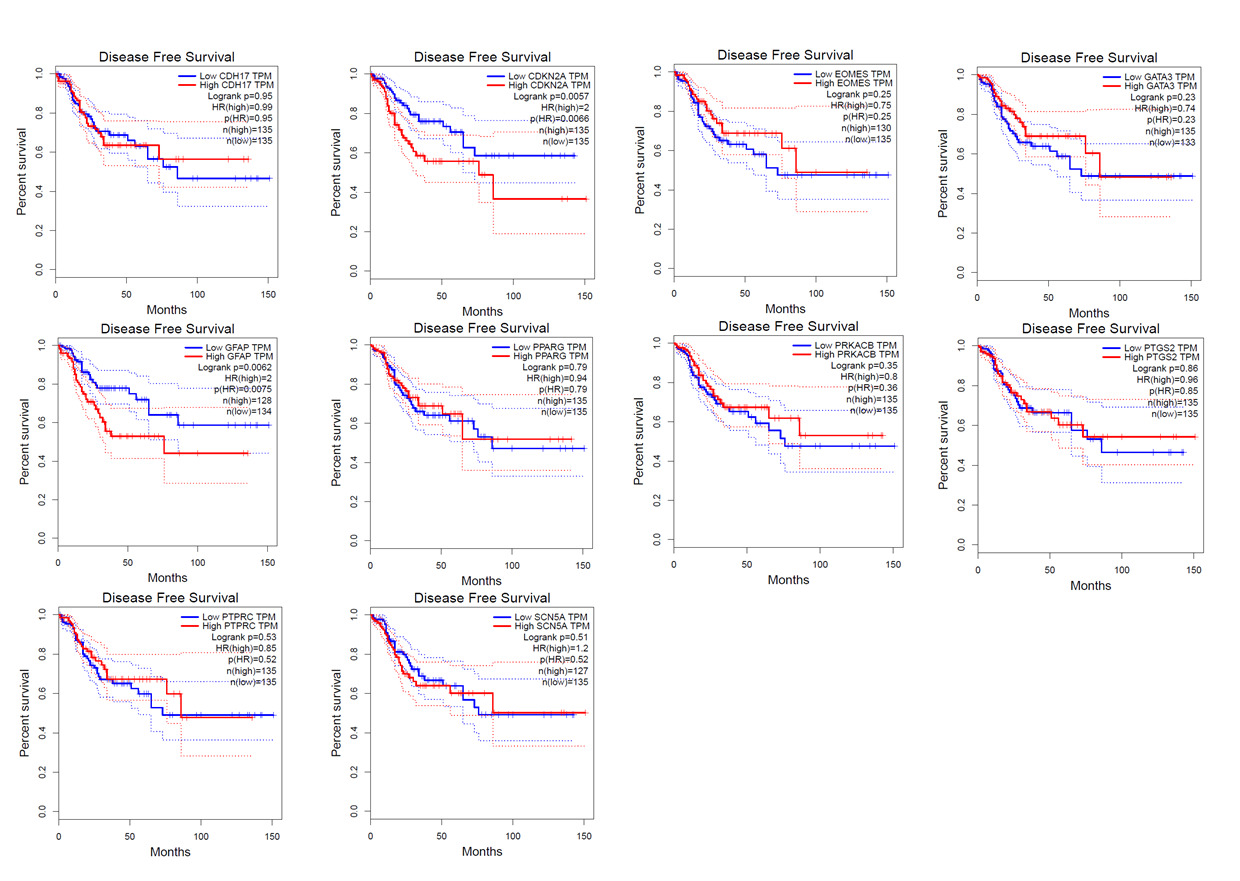

Supplement: Supplementary file 1 — Supplementary Figure S1. [file 41598_2022_11697_MOESM1_ESM.png]

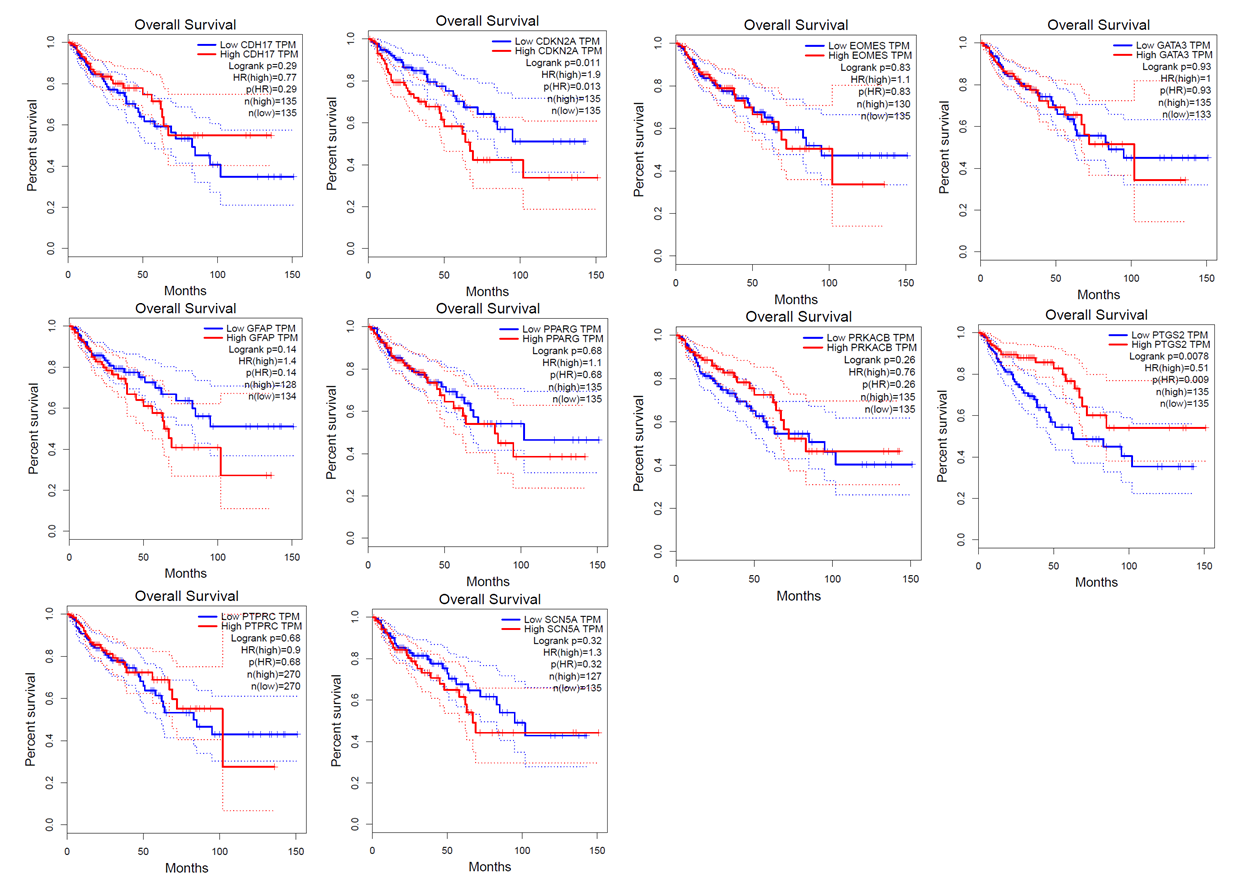

Supplement: Supplementary file 2 — Supplementary Figure S2. [file 41598_2022_11697_MOESM2_ESM.png]

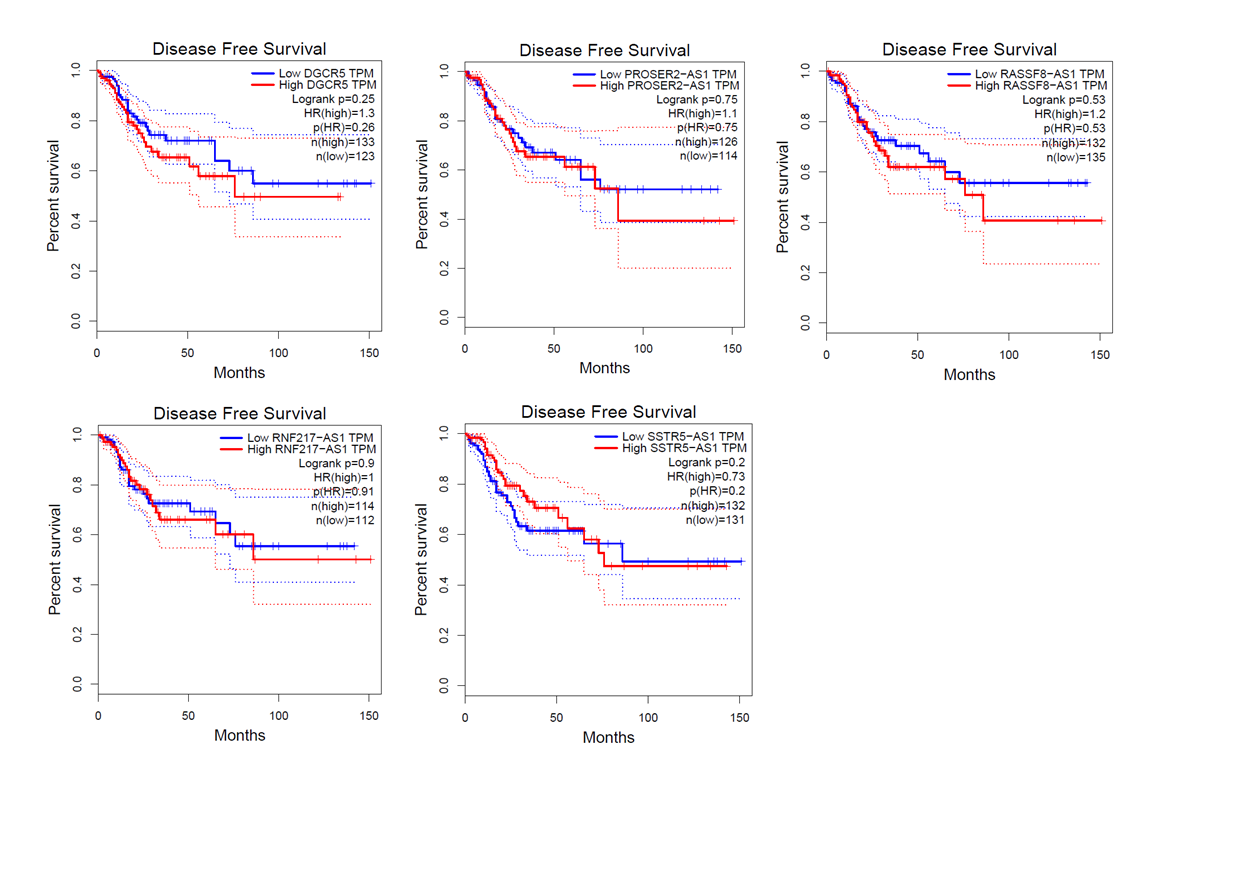

Supplement: Supplementary file 3 — Supplementary Figure S3. [file 41598_2022_11697_MOESM3_ESM.png]

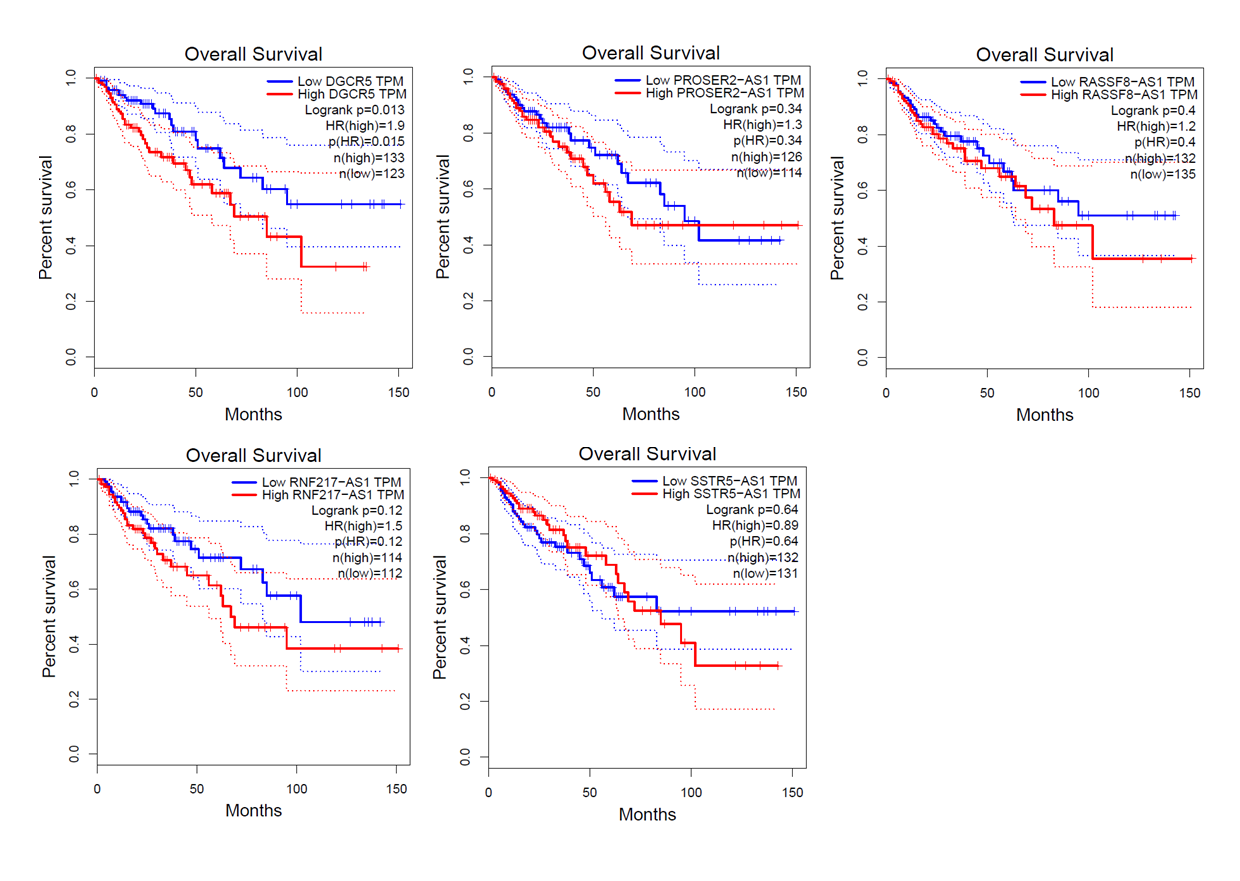

Supplement: Supplementary file 4 — Supplementary Figure S4. [file 41598_2022_11697_MOESM4_ESM.png]

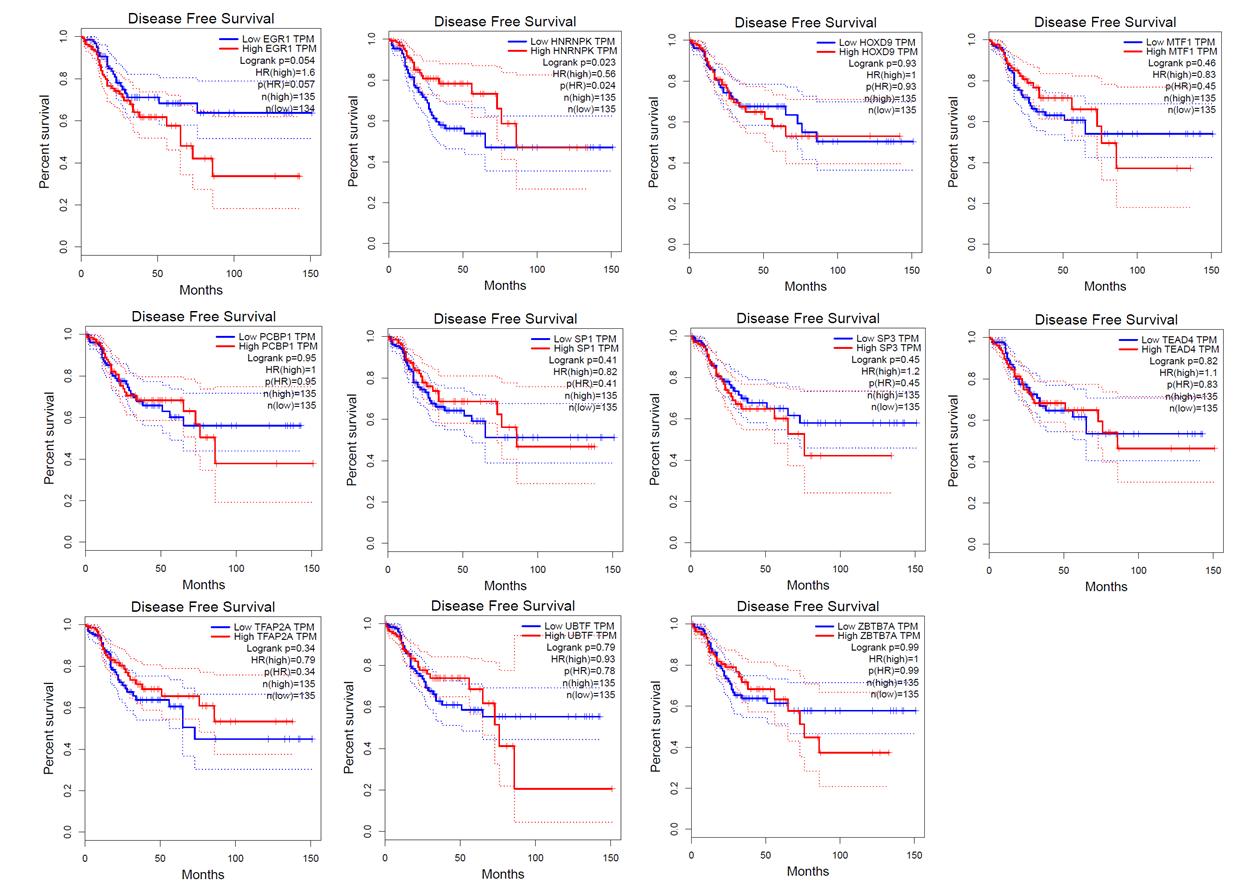

Supplement: Supplementary file 5 — Supplementary Figure S5. [file 41598_2022_11697_MOESM5_ESM.tif]

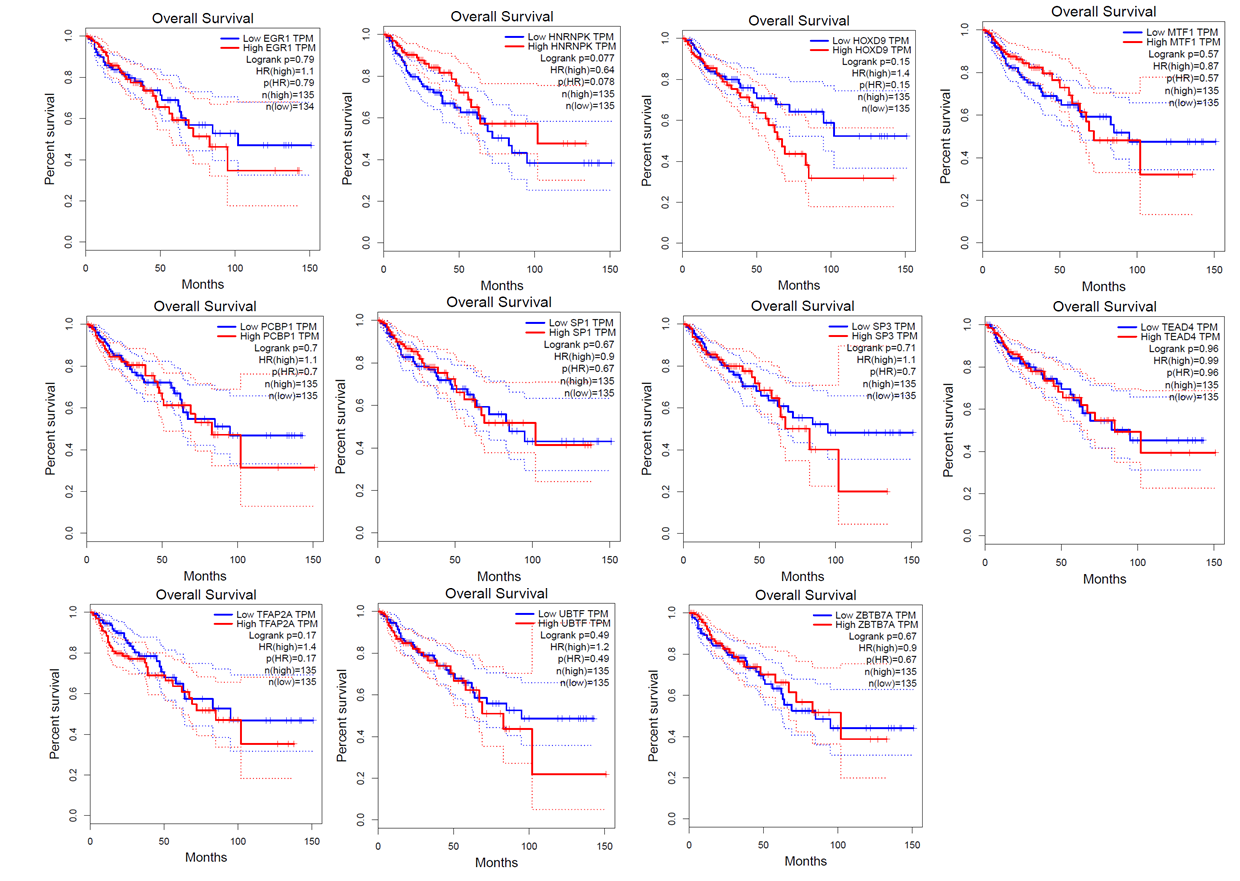

Supplement: Supplementary file 6 — Supplementary Figure S6. [file 41598_2022_11697_MOESM6_ESM.png]

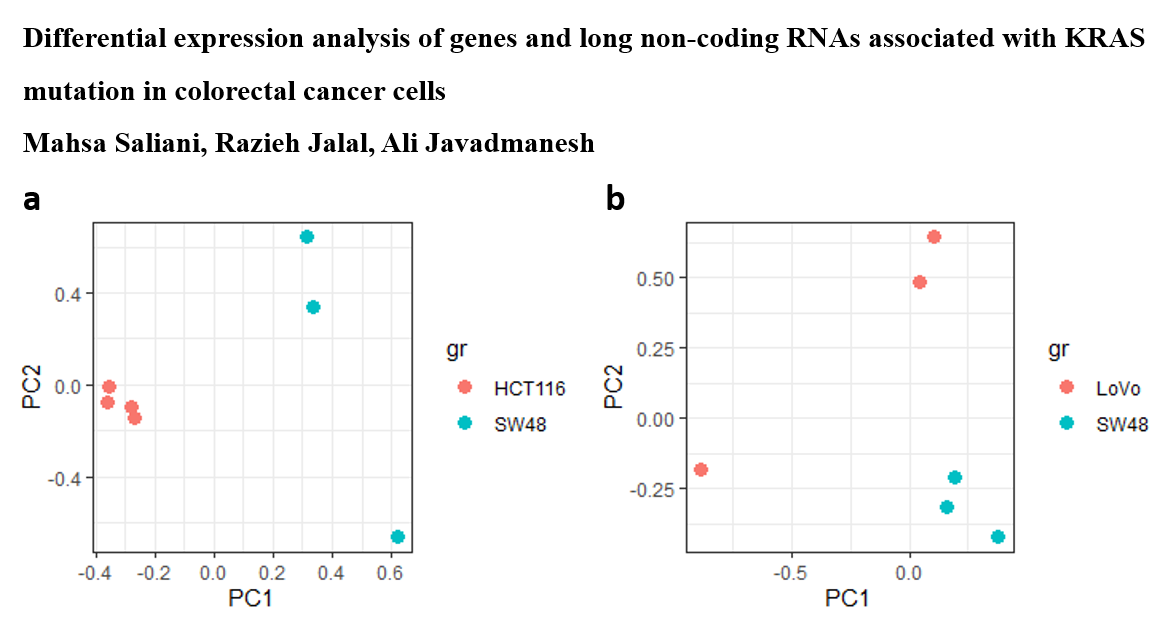

Supplement: Supplementary file 11 — Supplementary Data 4. [file 41598_2022_11697_MOESM11_ESM.png]
