# Supplementary material for: Differential expression analysis of genes and long non-coding RNAs associated with KRAS mutation in colorectal cancer cells
Source: Sci Rep. 2022 May 13;12:7965. doi: 10.1038/s41598-022-11697-5 (PMC9106686; doi:10.1038/s41598-022-11697-5)
Supplement: Supplementary file 7 — Supplementary Legends. [file 41598_2022_11697_MOESM7_ESM.docx]

**Supplementary Data legends**

**Supplementary Data 1.** Fold changes and adjusted p-value of DEGs in HCT-116 versus SW48. DEGs were defined as |log2FC| ≥ 1.5 and adjusted p-value < 0.05.

**Supplementary Data 2.** Fold changes and adjusted p-value of DEGs in LoVo versus SW48. DEGs were defined as |log2FC| ≥ 1.5 and adjusted p-value < 0.05.

**Supplementary Data 3.** Overlapped DEGs as KRAS-dependent gene expression signature.

**Supplementary Data 4**. Principal component analysis. The discriminating genes were used to generate PCA plot of the data. PCA multidimensional scaling visualization, based on the gene expression data and separated linearly CRCs with and without KRAS mutation. (a): PCA plot based on the gene expression data of HCT-116 versus SW48. (b): PCA plot based on the gene expression data of LoVo versus SW48. gr: group.

**Supplementary Figure legends**

**Figure S1.** Disease-free analysis of the hub genes in the prognosis of colorectal cancer. The Kaplan–Meier plot indicates the prognostic ability of hub genes in CRC tissues using GEPIA tool considering a statistical significance level at log-rank p < 0.05.

**Figure S2.** Overall survival analysis of the hub genes in the prognosis of colorectal cancer. The Kaplan–Meier plot indicates the prognostic ability of hub genes in CRC tissues using GEPIA tool considering a statistical significance level at log-rank p < 0.05.

**Figure S3.** Disease-free analysis of the lncRNAs in the prognosis of colorectal cancer. The Kaplan–Meier plot indicates the prognostic ability of lncRNAs in CRC tissues using GEPIA tool considering a statistical significance level at log-rank p < 0.05.

**Figure S4.** Overall survival analysis of the lncRNAs in the prognosis of colorectal cancer. The Kaplan–Meier plot indicates the prognostic ability of lncRNAs in CRC tissues using GEPIA tool considering a statistical significance level at log-rank p < 0.05.

**Figure S5.** Disease-free analysis of the TFs in the prognosis of colorectal cancer. The Kaplan–Meier plot indicates the prognostic ability of TFs in CRC tissues using GEPIA tool considering a statistical significance level at log-rank p < 0.05.

**Figure S6.** Overall survival analysis of the TFs in the prognosis of colorectal cancer. The Kaplan–Meier plot indicates the prognostic ability of TFs in CRC tissues using GEPIA tool considering a statistical significance level at log-rank p < 0.05.
