# Supplementary material for: Differential expression analysis of genes and long non-coding RNAs associated with KRAS mutation in colorectal cancer cells
Source: Sci Rep. 2022 May 13;12:7965. doi: 10.1038/s41598-022-11697-5 (PMC9106686; doi:10.1038/s41598-022-11697-5)
Supplement: Supplementary file 12 — Supplementary Table 1. [file 41598_2022_11697_MOESM12_ESM.docx]

**Supplementary Table 1:** GSEA analysis shows dysregulated oncogenic signatures in mtKRAS samples versus wtKRAS cell.

| **HCT-116 versus SW48** | | **LoVo versus SW48** | |
| --- | --- | --- | --- |
| **Upregulated signatures** | **Downregulated signatures** | **Upregulated signatures** | **Downregulated signatures** |
| KRAS.50_UP.V1_UP_15 | P53_DN.V1_DN_12 | E2F1_UP.V1_UP_188 | BCAT_BILD_ET_AL_DN_209 |
| KRAS.300_UP.V1_UP_6 | PTEN_DN.V1_DN_9 | KRAS.300_UP.V1_DN_134 | CRX_DN.V1_DN_149 |
| KRAS.600.LUNG.BREAST_UP.V1_UP_51 | RB_P107_DN.V1_DN_93 | KRAS.600.LUNG.BREAST_UP.V1_UP_152 | CYCLIN_D1_KE_.V1_DN_164 |
| KRAS.600_UP.V1_UP_18 | B_P130_DN.V1_DN_87 | KRAS.BREAST_UP.V1_DN_119 | P53_DN.V2_UP_137 |
| KRAS.PROSTATE_UP.V1_UP_33 | CTIP_DN.V1_DN_24 | KRAS.BREAST_UP.V1_UP_146 | PDGF_ERK_DN.V1_DN_182 |
| KRAS.KIDNEY_UP.V1_DN_66 | TBK1.DF_DN_102 | KRAS.DF.V1_UP_125 | PGF_UP.V1_DN_176 |
| ALK_DN.V1_UP_30 | GCNP_SHH_UP_EARLY.V1_DN_60 | KRAS.KIDNEY_UP.V1_UP_161 | PRC2_EED_UP.V1_DN_170 |
| BCAT_GDS748_UP_84 | DCA_UP.V1_DN_45 | KRAS.LUNG.BREAST_UP.V1_UP_113 | PTEN_DN.V1_DN_131 |
| HOXA9_DN.V1_UP_99 | PDGF_ERK_DN.V1_DN_81 | MYC_UP.V1_UP_185 | RB_P107_DN.V1_DN_200 |
| NOTCH_DN.V1_UP_36 |  | EIF4E_UP_173 | RB_P130_DN.V1_DN_194 |
| E2F3_UP.V1_UP_39 |  | GCNP_SHH_UP_EARLY.V1_UP_197 |  |
| ESC_V6.5_UP_EARLY.V1_UP_75 |  | GLI1_UP.V1_UP_203 |  |
| GLI1_UP.V1_UP_63 |  | LEF1_UP.V1_DN_107 |  |
| STK33_UP_69 |  | PDGF_UP.V1_UP_179 |  |
